# Use of the Pittsburgh Sleep Quality Index in People With Schizophrenia Spectrum Disorders: A Mixed Methods Study

**DOI:** 10.3389/fpsyt.2019.00284

**Published:** 2019-05-09

**Authors:** Sophie Faulkner, Chris Sidey-Gibbons

**Affiliations:** ^1^School of Health Sciences, University of Manchester, Manchester, United Kingdom; ^2^Greater Manchester Mental Health NHS Foundation Trust, Manchester, United Kingdom; ^3^Patient Reported Outcomes, Value & Experience Center (PROVE), Brigham and Women’s Hospital, Boston, MA, United States; ^4^Faculty of Surgery, Harvard Medical School, Boston, MA, United States

**Keywords:** screening, outcome measure, psychometric, interview, qualitative, psychosis, circadian rhythm disorder, insomnia

## Abstract

The Pittsburgh Sleep Quality Index (PSQI) is a measure of self-reported sleep quality and sleep disturbance. Though the PSQI is widely used, it is unclear if it adequately assesses self-reported sleep disturbance in people with schizophrenia spectrum disorders. We used mixed methods to examine the relationship between scores on the PSQI and qualitative self-report during in-depth interview in a group of participants diagnosed with schizophrenia spectrum disorders (N = 15). Although the PSQI appears to accurately capture issues related to sleep initiation, average duration, and interruption by physical complaints, it did not adequately assess other salient issues including irregularity in sleep duration and timing, shallow unrefreshing sleep, prolonged sleep inertia, hypersomnia, and sleep interrupted by mental or psychological complaints. In interview by contrast these types of problems were readily reported and described as important by participants. Our findings suggest that using the PSQI summary score as a measurement of general sleep disturbance in this population may be misleading, as this failed to capture some of the types of sleep problems that are particularly common in this group.

## Introduction

The Pittsburgh Sleep Quality Index (PSQI) is a widely used self-reported questionnaire measure of sleep (1). The PSQI is practical and brief, returning a single score representing overall sleep quality, which incorporates qualitative and quantitative aspects of sleep; scores above 5 are suggested as indicative of a potential sleep problem. The PSQI includes open-ended questions that can be used to identify the nature and possible causes of sleep problems to help direct treatment (2), and gives subscale scores that can indicate the type of sleep problems (sleep duration, latency, disturbances, quality, efficiency, daytime dysfunction, and use of sleep medication), as well as some questions on indicators of sleep apnea.

The PSQI was initially developed with a sample of people with depression, healthy sleepers, and people with sleep disorders (2). Many authors, including those who developed the PSQI, have subsequently developed new measures; some of which utilize more modern standards for patient involvement and psychometric analysis (1, 3). The widespread adoption of the PSQI has led to a large literature that appears to facilitate comparison between samples, and now acts as a motivating factor for researchers and clinicians to continue to use the PSQI in preference of other measures. This includes some populations for which it has not been validated (4).

The PSQI is often used as a global measure of self-reported sleep disturbance in people with schizophrenia spectrum disorders, but the validity of the PSQI (English version) as a global measure of sleep disturbance in this population has, to our knowledge, not been evaluated. We explored the content validity of the PSQI for assessing patient-reported sleep quality and sleep disturbance in people with schizophrenia spectrum disorders.

## Methods

We recruited 15 adults with schizophrenia spectrum disorders, who were in contact with specialist mental health services in the United Kingdom, and had a self-reported problem with sleep initiation, maintenance, quality, timing, or refreshingness. People whose predominant complaint was of parasomnia or sleep apnea were not included, in order to focus on insomnia and circadian rhythm problems. All participants’ data are included in the current analysis. Written informed consent was obtained after participants reviewed the participant information sheet and had sufficient opportunity for further explanation or questions. Ethical approval was obtained through the NHS Research Ethics Committee Proportionate Review Service (14/NS/1085).

Demographic data were collected, and symptoms and functioning were rated using the Global Assessment of Functioning (GAF) split version (GAF-F and GAF-S). Participants completed the PSQI and were invited to report their experience as they went through the questionnaire; this was followed by an in-depth interview that further explored their sleep experiences and complaints (see **Supplementary File S1**). In addition to the planned questions, the interviewer used continuers and active listening techniques; interview questions later went on to cover other research aims including exploring acceptability of potential treatments, and barriers to improving sleep; these results are reported elsewhere (5). The use of in-depth interview is an additional complementary approach to quantitative psychometrics in evaluating the validity of standardized self-report questionnaires, and it has been used informatively to examine interpretation of other self-reported health measures (6).

Transcription noted long pauses and we made field notes on nonverbal expressions (e.g., frowning, and nonlexical utterances such as “Errrr…”). Analysis was facilitated by Nvivo (qualitative analysis software), creating an audit trail, and ensuring themes were linked back to the data. Transcript content was coded in relation to the PSQI question each section related to, and in relation to emergent themes and subthemes from in-depth analysis of participant interviews. The qualitative analysis used a framework approach, and samples of the data were independently analyzed by a second researcher to enhance reliability. The mixing of methods was approached through qualitization of individual PSQI component scores, and summarizing and classification of the sleep problems described in interviews, to facilitate comparison of component and total PSQI scores, with the clinical presentation as described in each participant’s qualitative account (7).

## Results

Fifteen participants were recruited, including acute inpatients (n = 2), outpatients receiving intensive (daily) support (n = 3), those under Community Mental Health Teams (n = 8), and under outpatient clinic only (n = 2); none were currently in paid employment. There were 10 males and 5 females, with a mean age of 45.9 (SD = 10.55), diagnosed with schizophrenia (n = 8), schizoaffective disorder (n = 6), and delusional disorder (n = 1). All were prescribed either one (n = 8) or two antipsychotics (n = 7); dosages as a fraction of defined daily doses (8) ranged from 0.36 to 5.5 (mean 2.19, SD 1.28). GAF-F and GAF-S scores ranged from 38 to 85, and from 21 to 80, respectively (mean 62.3, SD 12.16 and mean 47.7, SD 19.40, respectively). For itemized PSQI component scores and raw values, see **Table 1**. Overall, the areas in which these participants scored higher (indicating worse problems) were sleep latency, sleep quality, and daytime dysfunction, while sleep duration scored very low (which should suggest minimal problems). **Table 2** shows summary statements describing the nature of sleep complaints described in interview, and a breakdown of participants endorsing various types of problems.

**Table 1:** Participant itemized PSQI scores, hours of sleep, and minutes sleep latency.

Respondent	Hours of actual sleep: h/score^1^	Sleep latency: min/score^1^	Sleep disturbances^1^	Daytime dysfunction^1^	Habitual sleep efficiency^1^	Self-reported quality^1^	Use of sleeping medication^1^	Total PSQI score^2^
r01	11/0	60/3	0	0	0	0	0	**3**
r02	4.5/3	3/0	1	2	1	2	3	**12**
r03	12/0	10/0	1	2	0	0	3	**6**
r04	6/1	60/1	0	0	0	0	0	**2**
r05	14.5/0	180/3	1	3	0	1	0	**8**
r06	6.5/1	60/3	1	3	1	1	0	**10**
r07	4/3	120/3	1	1	2	2	3	**15**
r08	8.5/0	20/1	1	1	0	1	3	**7**
r09	11/0	30/2	2	1	1	1	0	**7**
r10	4.5/3	30/2	1	3	3	3	3	**18**
r11	8/0	30/1	1	0	1	1	0	**4**
r12	3/3	180/3	1	2	3	1	0	**13**
r13	8/0	60/3	1	0	0	2	3	**9**
r14	9/0	10/2	1	0	0	0	0	**3**
r15	7/0	60/3	2	2	1	2	3	**13**
**Averages**	7.83/0.9	61/2.0	1.0	1.3	0.9	1.1	1.4	**8.7**
Number of participants PSQI indicates have poor sleep quality = 11								

^1^Minimum score = 0 (better), maximum score = 3 (worse). ^2^Minimum score = 0 (better), maximum score = 21 (worse), Total >5 associated with poor sleep quality. PSQI, Pittsburgh Sleep Quality Index.

**Table 2:** Qualitative summaries of participant’s description of their sleep problems.

Self-report regarding sleep	r01	r02	r03	r04	r05	r06	r07	r08	r09	r10	r11	r12	r13	r14	r15
Frequent problem with long sleep latency^1^				x			x					x	x		x
Occasional severe problem with long sleep latency^1^	x	x									x				
Problem maintaining sleep or excessively early rising^1^		x			x	x	x			x		x	x		
Problem with difficulty rising or waking^1^	x			x	x				x					x	x
Problem with too long sleep duration^1^			x		x					x				x	
Unusually long sleep, not a problem at present^2^	x								x						
Usually regularly naps^2^	x				x	x			x	x	x	x			x
Problem with prolonged sleep inertia^1^		x	x	x	x	x			x					x	
Significantly troubled by bad dreams^1^		x						x		x		x	x		x

1 Items are selected where this was a major and consistent aspect of the participant’s self-reported complaint; items are only selected where the participant subjectively perceived this as a problem.

2 Items are selected where this was a frequent occurrence, may not subjectively be a problem.

### Acceptability

Most participants reported no problem completing the measure and felt it asked relevant questions. Although some noted a preference for open questions, this is not an issue specific to the PSQI. A minority of participants suggested there should be more questions relating to psychological or mental health-related causes of sleep disruption.

### Sleep Duration

Nine participants scored 0 for sleep duration; however, four of these participants were sleeping 11 to 14.5 h, and expressed concerns about this:

“I’ve looked it up on Google and it says things like, a higher risk of heart attack, diabetes or early death [laughs]. [ … ] I do feel guilty, quite a lot, yeah. I would like to be able to … I would love to be able to just be a morning person” (r05, reported sleep duration = 14.5 h, score on this item = 0)

Hypersomnia was seen as a potential health concern, and a cause for negative self-concept:

“you’re lazy … you’re wasting your life away” (r12).

Participants who described a large amount of nightly variation found average sleep duration difficult to calculate accurately:

“sometimes [waking up] will be 6 o’clock for like three weeks, but very rarely it’s 11 o’clock [ … ] if I’m feeling too enthusiastic I’ll be awake all night because of the excitement from the day [ … ] yeah so it asks for a fixed time, but it’s quite hard to estimate, it varies a lot” (r02, reported average sleep duration = 4.5 h, score on this item = 3)

Seven of the 15 participants tried to give ranges, which were sometimes several hours apart; three participants described nights of getting almost no sleep at all, followed by very long sleep when they did eventually sleep. These participants’ mean total sleep time did not capture this issue as the very long and very short sleep times were averaged to a normal duration. Some distinguished “actual sleep” from light sleep/partial sleep, so were unsure how best to respond. Some answers weren’t internally consistent (e.g., time to bed till time to wake, minus sleep latency and sleep interruptions, was inconsistent with reported total sleep time). It should be acknowledged that the latter of these issues could affect many retrospective self-report measures.

It is important also to note that daytime naps are not included within the sleep duration total, and for over half in this sample (eight participants), naps were a significant source of sleep:

“Respondent: [ … ] I always go to sleep in the afternoon

Interviewer: Most days?

Respondent: Yeah I’d say every day.

Interviewer: How long for?

Respondent: One and a half to two hours” (r10, reported sleep duration = 4.5 h, score on this item = 3)

Other participants described 3- or 4-h naps; hence, the PSQI total sleep time question missed roughly half of their actual total sleep for that day.

### Sleep Timing

Average sleep timings are measured by the PSQI, but do not contribute directly to the score. The times recorded give useful information for clinicians on circadian preference or social commitments when sleep timing is regular; in this group, however, times varied and averages may be less meaningful. This variability may have been missed altogether if times were averaged by participants without comment. Dissatisfaction with sleep timing, and unpredictability of sleep timing, were significant sources of dysfunction and distress in participant accounts; participants described the impact on their ability to take on work, education, or social commitments. Satisfaction with or regularity of sleep timing is not part of the PSQI scoring so a problem with sleep timing does not directly impact the score.

### Sleep Latency

Sleep latencies reported in answering the PSQI and in interview were similar. While it is interesting that some participants had long sleep latency (60 min, score 3 = worst) and were untroubled by this, this constitutes a quantitative aspect of their sleep disturbance, which is accurately summarized by the PSQI.

### Sleep Disturbance

It is not clear how decisions were made regarding what are considered normal frequencies for some of the listed types of sleep disturbance, resulting in floor and ceiling effects. Participants who described “problems” with going to the bathroom at night went several times per night, the highest option to select being ×3 per week. Someone who woke to use the toilet twice a week explained that they felt this was probably less than most people. With regards to the impact of bad dreams, it seemed that frequency of bad dreams bore limited relation to the distress caused; in this sample, no one endorsed 3× per week, but some described less frequent but intense bad dreams causing significant distress, which was not well captured:

“Oh jeeze I’m always having bad dreams … I’d say maybe twice a week.” (r12)

Significantly, in interview the vast majority of the sample complained of problems with poor sleep maintenance and depth, in terms of “broken” sleep, sleep that was “not deep,” or was not “proper sleep”; however, the average score for sleep disturbance was 1.0 (0 = best, 3 = worse). This can largely be attributed to the aggregation of scores from physical and psychological causes of sleep disturbance where there are more physical causes listed. It was noted that sleep, which was disturbed by a wide range of causes, scored higher than sleep very often disturbed, but generally by the same cause. Comparison of individual accounts and sleep disturbance scores showed that sleep that felt broken, but without a complete awakening, did not readily translate to a high score for “sleep disturbance.”

### “Another Reason…”

Although perhaps a trivial matter, the phrasing of this question proved problematic for well over half of participants, causing a pause in completion and questions regarding either the first part or both parts of this question:

“…but what does that mean, how often during the past month have you had trouble sleeping because of this, because of what?” (r10)

It was also noted that this question rarely elicited information, as participants brought up many “other reasons” in interview (e.g., hypnopompic hallucinations), but wrote “N/A” on the PSQI. This may contribute to the unusually low scores for sleep disturbance, as these “other reasons” were not scored.

### Daytime Dysfunction

The PSQI asks about trouble staying awake “while driving, eating meals, or engaging in social activity”; this question appeared not to be sensitive to sleepiness in those who did not drive and socialized infrequently:

“My sleep’s a bit spontaneous, my body don’t plan it [ … ] I’m always falling asleep watching films [ … ] but no, I’m trying to think now how often I do a social activity, it’s less than once a week isn’t it. So it’s less than once a week isn’t it falling asleep.” (r15, score on this item = 0)

Many asked if falling asleep in front of the television counted, and some then answered yes for this question.

The other question concerns “enthusiasm to get things done”; some noted they had difficulty with enthusiasm due to mood or psychotic symptoms, rather than their sleep. Daytime dysfunction questions did not pick up on the impact on daytime functioning, where participants were napping or sleeping for excessive periods to counter tiredness:

“I’m really fed up because I’d like to get out and do things instead of sleeping the days away” (r01, daytime dysfunction score = 0)

“It can be as short as half an hour or as long as two hours [ … ] I go to sleep because I’m tired in the day time, not because I’m bored.” (r14, daytime dysfunction score = 0)

Sleep inertia and difficulty waking were also major complaints discussed by participants within the current study (n = 6 of 15). These should be detected as a form of daytime dysfunction; however, half of those expressing this complaint scored 0 for daytime dysfunction (see **Tables 1** and **2**).

### Sleep Efficiency

Sleep efficiency has been found to be lower on average in groups with schizophrenia (9); in this sample, average sleep efficiency score does not suggest that this was a particular difficulty (average = 0.9). Interview accounts described difficulties initiating sleep, or maintaining sleep, suggesting poor sleep efficiency. Of those who reported one or more of these difficulties (n = 11), the average sleep efficiency score was 1.09; only four reporting such complaints scored 0. This suggests that the sleep efficiency score detected relevant problems to some extent in most cases.

### Quality

Interestingly, over half of participants rated quality as very good (0) or fairly good (1), but went on to state significant concerns with their sleep and its impact on their life, including that their sleep was not restorative, that their poor sleep pattern was a barrier to getting a job, or that their sleep was “medicated sleep” and therefore substandard:

“[If I wasn’t on medication] That I’d actually sleep, yeah. And I think I’d be able to do more things as well, you know, in the day, if I wasn’t on the medication, sometimes, if I managed to get natural sleep.” (r03, sleep quality rated “very good” = 0)

For some, there seemed to be a direct contradiction between self-reported “sleep quality” on the PSQI and their view of their sleep during interview:

“Interviewer: …how would you describe your sleep, if you were sort of telling someone about it?

Respondent: Umm, not very good.

Interviewer: No?

Respondent: No. Not like other people … go on like sleep’s supposed to be, you know?

Interviewer: What?

Respondent: Like, when they say have a nice sleep and you’ll feel refreshed and all this nonsense.

Interviewer: They say that.

Respondent: Yes, and I don’t feel like that.

Interviewer: No.

Respondent: I feel like, jeez, what’s happened?” (r12, sleep quality rated “fairly good” = 1)

In the context of the rest of the analysis (5), this can be attributed to lowered expectations, so “fairly good” could mean good—when all is considered, good—compared to others with the same condition. It is also possible that participants responded regarding sleep quality by evaluating individual periods of sleep obtained, in contrast to the adequateness of their day-to-day sleep as a whole. Potentially also more rapport was built during the in-depth interview and participants felt more open and prepared to describe problems.

### Sleeping Medication

The present sample’s highest scoring domain was use of sleeping medication. This did not, however, represent high levels of hypnotic use in this sample; in six out of seven of those scoring 3 (highest), their answer related to their oral antipsychotic being “sleeping medication” (although some felt this was an ineffective sleeping medication). Some described in interview using their antipsychotic to control their sleep onset, but answered “never” to this question on the PSQI, and some raised the dilemma of whether their antipsychotic counted or not. Answers were therefore dictated by semantic interpretation rather than any meaningful differences between perceptions or behaviors.

### Sleep Disordered Breathing

The PSQI includes questions regarding snoring or breathing among its sleep disturbance questions, and also a section for completion by the person’s bed partner/roommate to screen for sleep disordered breathing, in acknowledgement of people’s reduced awareness of their own breathing during sleep. Participants in this study did endorse snoring, but rarely endorsed “cannot breathe comfortably,” rather clarifying that they breathed heavily, not had *difficulty* breathing:

“…cause I’m a big lad as well so when I’m lying down … I’d say not it’s hard to breathe but I breathe heavily.” (r15, cannot breathe comfortably = 1, cough or snore loudly = 3, circled ‘loudly’ for emphasis).

It was never designed as such, and it is important that the PSQI is not considered to be an effective screening for sleep disordered breathing, particularly without the bed partner/roommate questions being completed.

### Total Scores

The total PSQI scores indicated that seven participants were either good sleepers or had only mild sleep problems (n = 4 score <5, n = 3, score 6–7). Of these seven, four described significant and severe concerns during interview, while the other three described milder but definite problems. Of those whose PSQI scores suggested moderate or severe problems (score 8–18), the global impression from the interview was also of moderate or severe problems.

## Discussion

The sample reported multiple and complex problems with sleep initiation, continuity, quality, and timing, with attendant daytime dysfunction; the PSQI was capable to assess some, but not all, of these issues. The PSQI appeared to be suitable for identifying self-reported short sleep, long sleep latency, or complete awakenings during the night, but poorly represented some other problems such as variable and inconsistent sleep length, poor sleep depth or quality, increased sleep inertia, hypersomnia, and inappropriate or inconsistent sleep timing. While issues with sleep timing are beyond the intended scope of the PSQI, sleep duration, quality, and daytime dysfunction are within its scope and were poorly captured. Furthermore, it is common for total PSQI scores to be treated as a global measure of sleep disturbance, which these findings suggest is not valid. Reliance on total PSQI score as a measure of sleep dysfunction is particularly inappropriate for those with schizophrenia spectrum disorders whose sleep problems include more circadian dysregulation than other groups (10), and who as a result experience more inconsistent and variable sleep, and more difficulties timing sleep patterns to fit with life expectations (5, 11, 12). Some of the measurement issues highlighted also have potential implications for interpretation of PSQI scores in other populations.

### Measuring Sleep Duration, Variability, and Depth

As some participants feared, both excessively short and excessively long sleep are indeed associated with increased mortality (13), and it has previously been recommended that the relationship of sleep duration to assumed sleep quality on the PSQI should be U-shaped and not linear (14). Our findings support this suggestion, concurring that unusually long sleep, as well as too short sleep, caused concerns for participants. It is also important to note that in people taking significant naps, as was common in this sample, the PSQI can mischaracterize (underestimate) a person’s total sleep time, as might also occur in regional populations in whom biphasic sleep is common. These issues with calculation and scoring of sleep duration of course affect the use of the PSQI in many other clinical and nonclinical samples, not just in those with schizophrenia spectrum disorders.

Participant PSQI scores were similar to those from research with the Japanese version of this instrument in a similar sample (14), where sleep latency, sleep quality, and daytime dysfunction received higher scores on the PSQI, while sleep duration scored very low [which should suggest minimal problems; Doi et al. (14) also noted hypersomnia was not captured]. In contrast to our study, the subgroup of the Japanese sample with schizophrenia (n = 24) scored low regarding sleeping medication, perhaps owing to different phrasing in the translation (14).

Insufficient detection of problems with sleep depth is significant particularly for this population, for whom levels of shallow sleep (stage 1) are often elevated, and deeper sleep (Stage 2 and Stage 3 non-REM sleep) is often reduced (9). Although objective assessment of sleep depth requires polysomnography or spectral analysis, the experience of deep sleep was important to participants. Even apart from importance to individuals, subjective evaluations of sleep have often been found to be equally if not more predictive of health and functioning outcomes than some more objective measures (15, 16), suggesting even “inaccurate” experiences may be equally important to capture.

Our findings are consistent with those of Waters et al. (12) who found only small and statistically nonsignificant differences in PSQI scores between people with schizophrenia and healthy controls, but found increased variability in sleep latency, efficiency, and duration in schizophrenia when using actigraphy. Actigraphy or sleep diaries can be recommended to assess variability. However, retrospective self-report is less burdensome and has the potential to offer some insight; a future measure might include questions that assess how frequently various sleep values deviate from the average, by more than a certain amount (e.g., “How many times in the last month? was it 2 h more or less than this?”). Appropriate phrasing, format, and content would require development and testing.

### Clinical Assessment of Sleep

For current clinical practice, supplementary questions or additional measures should be used when using the PSQI as a screening for sleep problems. For instance, the PSQI should not be relied upon to screen for sleep disordered breathing; a ready alternative is the STOP-Bang questionnaire, which has been found to be reasonably accurate in detection (17) and is freely available and brief (18). Measures of circadian preference might be added (19, 20); however, these do not measure regularity of rhythm. It is possible to measure and quantify regularity of rest-activity rhythms through actigraphy, describing both amplitude (relative amplitude) and regularity of rhythm (interday stability) (21), as has been more extensively utilized in samples with dementia (22) who also experience circadian dysregulation. At least one retrospective self-report measure of regularity is available [e.g., (23)], although none has yet been tested in schizophrenia spectrum disorders. During clinical interview, therefore, additional questions are recommended regarding regularity of sleep timing, and the match between sleep timing and individual lifestyle choices and requirements.

### Outcome Measurement

Whether for research or clinical outcome measurement for quality improvement, the findings of the present study caution against relying on the PSQI total score alone, as improvements in sleep timing or regularity (often accompanied by improved quality of life and functioning) may go undetected. There are more recently developed tools, including the PROMIS sleep dysfunction item bank (1), and the Glasgow Sleep Impact Scale (3), which have been specifically designed to act as a barometer of the patient’s perceived standard of sleep. Both tools were developed with patient involvement and have undergone validation in healthy controls and those with sleep disorders, or in insomnia, respectively. These measures are very promising and may offer a useful adjunct to clinical assessments of sleep issues for people with schizophrenia spectrum disorders, and, by virtue of their use of modern psychometric methods, may also offer a reliable means of comparison across diagnostic groups.

These tools, however, are designed to measure change for research or clinical outcome measurement; they do not simultaneously help to characterize the sleep problem—as might be desired by a clinician. And in this respect, they do not replace the PSQI. Asking the patient to specify sleep latency, sleep times, and causes of sleep disturbance, as the PSQI does, can help identify the problem and therefore direct treatment. Unfortunately, the PSQI alone is likely to give an incomplete and sometimes misleading picture, in the case of people with schizophrenia spectrum disorders, and possibly many other groups.

### Limitations and Future Directions

The generalizability of the findings from a small sample might be questioned, although as the types of problems described are similar to those found in larger samples studied using quantitative methodologies, we believe these findings are transferable. Although the diversity in the type and extent of problems is potentially representative of the diverse problems experienced in this group, it also limits the number of cases with each particular type of problem (e.g., with short sleep, or with hypersomnia). Furthermore, diversity in the environmental context of the participants, particularly the inclusion of both inpatients and outpatients, complicates interpretation. It would also have been useful to find out the approximate length of time since diagnosis, and length of time on antipsychotic medication, to better describe the sample and facilitate comparison with other studies. In hindsight, it would also have been useful if participants had been asked to comment on the recommended interpretation of their component or total PSQI scores (for instance, “This score suggests overall you have good/slightly disturbed/severely disturbed sleep. Do you agree?”), which might have provided a further point of reference.

This study did not set out to make statistical analysis of sensitivity or specificity, but these findings suggest a hypothesis of too low sensitivity, and missed cases, but no issues with overdetection. Future studies comparing PSQI to other measures such as actigraphy or polysomnography might confirm or quantify this, and could further examine which types of problem are underdetected in a larger sample. More detailed description of the weaknesses this study has highlighted would support improved interpretation of the considerable body of important work, which has used the PSQI to describe their sample or to measure change.

Our analysis demonstrates that the PSQI is insensitive to some sleep issues, which are described as important to people with schizophrenia spectrum disorders. This disparity between the range of issues highlighted in our interviews and those covered by the PSQI may suggest a need for a disease-specific measure to achieve high sensitivity to the particular problems of this group. Another possibility is the development of a measure of sleep disturbance, which can equally measure sleep problems of circadian, insomniac, or combined cause and nature, which could be used trans-diagnostically. A future measure could attempt also to take into account different environmental contexts; for instance, in many institutional settings, patients may more commonly go “to bed” far in advance of intending to sleep, as their bedroom may be the only private space in which to wind down for sleep. This can lead to underestimation of sleep efficiency, as has previously been noted in relation to sleep diaries (24); and alternative phrasing around “into bed” has been recommended. There may also be utility in a measure that examines or considers the impact of a mismatch between environment, occupational routine, and the individual, upon sleep, as well as factors that are more inherent to the person.

### Self-Report Items versus Self-Evaluated Items

The PSQI is of course not unique among self-report measures in being affected by participants calibrating some of their responses in relation to their own context and peer group, as we found here regarding rating of sleep quality. Similar findings of peer group-dependent evaluations were presented by Adamson et al. (6) regarding the evaluation of general health:

“Mrs K: Oh, I suppose for my age my health is excellent., I mean to say, it wasn’t until I went up for the assessment I knew there was anything wrong with my heart” [(6), p142]

This context-dependent evaluation may equally measure a difference in a person’s perceived peer group, as much as based on a change in self-perceived sleep, and makes it difficult to use exclusively self-evaluated items to compare between populations. It may therefore be desirable to include some quantitative self-report elements, which are more influenced by the individual’s perception of their sleep than contextual factors.

## Conclusion

Future research should develop a valid and reliable tool, with a similar shared utility for both clinicians and researchers as the PSQI has uniquely offered; this shared utility no doubt facilitates understanding between clinicians and researchers, and accounts for its enduring popularity. The authors suggest the development of a new measure that can act as a clinical screening and initial interview, and as an outcome measure in research.

## Ethics Statement

Ethical approval was obtained through the NHS Research Ethics Committee Proportionate Review Service (14/NS/1085), North of Scotland Research Ethics Committee 1. Written informed consent was obtained after participants reviewed the participant information sheet and had sufficient opportunity for further explanation or questions. A disclosure or risk protocol and a distress protocol were followed during data collection, and information sharing with care providers was discussed with participants in advance (information was shared on participant’s request, or if any immediate risks necessitated information sharing).

## Author Contributions

SF designed and conducted the study, collected and analyzed the data, and wrote the first draft of the manuscript. SF and CS-G were involved in editing, conceptual formulation, and discussion of the findings and implications of the study.

## Funding

This work was supported by the National Institute of Health Research (NIHR), through funding support received independently by SF and CS-G during completion of this study. The NIHR had no direct involvement in study design, conduct, or dissemination.

## Conflict of Interest Statement

The authors declare that the research was conducted in the absence of any commercial or financial relationships that could be construed as a potential conflict of interest.

## Supplementary Material

File S1 Question schedule and optional prompts.

## References

[1] Buysse DJ Yu L Moul DE Germain A Stover A Dodds NE Development and validation of patient-reported outcome measures for sleep disturbance and sleep-related impairments. Sleep (2010) 33(6):781–92 [online]. Available from: https://www.ncbi.nlm.nih.gov/pmc/articles/PMC2880437 10.1093/sleep/33.6.781 PMC2880437 20550019

[2] Buysse DJ Reynolds CF 3rd Monk TH Berman SR Kupfer DJ The Pittsburgh Sleep Quality Index: a new instrument for psychiatric practice and research. Psychiatry Res (1989) 28(2):193–213. [online]. Available from: http://www.ncbi.nlm.nih.gov/pubmed/2748771 10.1016/0165-1781(89)90047-4 2748771

[3] Kyle SD Crawford MR Morgan K Spiegelhalder K Clark AA Espie CA The Glasgow Sleep Impact Index (GSII): a novel patient-centred measure for assessing sleep-related quality of life impairment in insomnia disorder. Sleep Med (2013) 14(6):493–501. 10.1016/j.sleep.2012.10.023 23347908

[4] Garrow AP Yorke J Khan N Vestbo J Singh D Tyson S Systematic literature review of patient-reported outcome measures used in assessment and measurement of sleep disorders in chronic obstructive pulmonary disease. Int J Chron Obstruct Pulmon Dis (2015) 10:293–307. [online]. Available from: http://www.scopus.com/inward/record.url?eid=2-s2.0-84922743032&partnerID=tZOtx3y1 25709424 10.2147/COPD.S68093 PMC4330032

[5] Faulkner S Bee P Experiences, perspectives and priorities of people with schizophrenia spectrum disorders regarding sleep disturbance and its treatment: a qualitative study. BMC Psychiatry (2017) 17(1):158 [online]. Available from: DOI 10.1186/s12888-017-1329-8 10.1186/s12888-017-1329-8 28464848 PMC5414297

[6] Adamson J Gooberman-Hill R Woolhead G Donovan J ‘Questerviews’: using questionnaires in qualitative interviews as a method of integrating qualitative and quantitative health services research. J Health Serv Res Policy (2004) 9(3):139–45 [online]. Available from: http://www.ncbi.nlm.nih.gov/pubmed/15272971 [Accessed November 4, 2014]. 10.1258/1355819041403268 15272971

[7] Migiro SO Magangi BA Mixed methods: a review of literature and the future of the new research paradigm. Afr J Bus Manage (2011) 5(10):3757–64 [online]. Available from: http://www.academicjournals.org/AJBM

[8] WHO WHO Collaborating Centre for Drug Statistics Methodology., p. ATC/DDD Index. [online]. Available from: https://www.whocc.no/atc_ddd_index/ [Accessed March 12, 2017].

[9] Chan MS Chung KF Yung KP Yeung WF Sleep in schizophrenia: a systematic review and meta-analysis of polysomnographic findings in case-control studies. Sleep Med Rev (2017) 32:69–84. [online]. Available from .org/10.1016/j.smrv.2016.03.001 10.1016/j.smrv.2016.03.001 27061476

[10] Pritchett D Wulff K Oliver PL Bannerman DM Davies KE Harrison PJ Evaluating the links between schizophrenia and sleep and circadian rhythm disruption. J Neural Transm (2012) 119(10):1061–75. 10.1007/s00702-012-0817-8 22569850

[11] Waite F Evans N Myers E Startup H Lister R Harvey AG The patient experience of sleep problems and their treatment in the context of current delusions and hallucinations. Psychol Psychother: Theory Res Pract (2015) 89:181–93 [online]. Available from: http://doi.wiley.com/10.1111/papt.12073 10.1111/papt.12073 PMC4879509 26285922

[12] Waters F Sinclair C Rock D Jablensky A Foster RG Wulff K Daily variations in sleep-wake patterns and severity of psychopathology: a pilot study in community-dwelling individuals with chronic schizophrenia. Psychiatry Res (2011) 187(1–2):304–6 [online]. Available from: http://www.ncbi.nlm.nih.gov/pubmed/21272939 [Accessed October 28, 2014]. 10.1016/j.psychres.2011.01.006 21272939

[13] Cappuccio FP D’Elia L Strazzullo P Miller MA Sleep duration and all-cause mortality: a systematic review and meta-analysis of prospective studies. Sleep (2010) 33(5):585–92 [online]. Available from: http://www.pubmedcentral.nih.gov/articlerender.fcgi?&artid=2864873tool=pmcentrez&rendertype=abstract 10.1093/sleep/33.5.585 PMC2864873 20469800

[14] Doi Y Minowa M Uchiyama M Okawa M Kim K Shibui K Psychometric assessment of subjective sleep quality using the Japanese version of the Pittsburgh Sleep Quality Index (PSQI-J) in psychiatric disordered and control subjects. Psychiatry Res (2000) 97:165–72. 10.1016/S0165-1781(00)00232-8 11166088

[15] Harvey AG Tang NKY (Mis)perception of sleep in insomnia: a puzzle and a resolution. Psychol Bull (2012) 138(1):77–101. [online]. Available from: http://www.pubmedcentral.nih.gov/articlerender.fcgi?artid=3277880&tool=pmcentrez&rendertype=abstract [Accessed October 22, 2014]. 10.1037/a0025730 21967449 PMC3277880

[16] Pilcher JJ Ginter DR Sadowsky B Sleep quality versus sleep quantity: relationships between sleep and measures of health, well-being and sleepiness in college students. J Psychosom Res (1997) 42(6):583–96. 10.1016/S0022-3999(97)00004-4 9226606

[17] Abrishami A Khajehdehi A Chung F A systematic review of screening questionnaires for obstructive sleep apnea. Can J Anaesth (2010) 57(5):423–38. 10.1007/s12630-010-9280-x 20143278

[18] Chung F Subramanyam R Liao P Sasaki E Shapiro C Sun Y STOP-Bang Questionnaire. Toronto: Toronto Western Hospital (2018). [online]. Available from: http://www.stopbang.ca/osa/screening.php [Accessed August 1, 2018].

[19] Roenneberg T Wirz-Justice A Merrow M Life between clocks: daily temporal patterns of human chronotypes. J Biol Rhythms (2003) 18(1):80–90. 10.1177/0748730402239679 12568247

[20] Randler C Díaz-Morales JF Rahafar A Vollmer C Morningness–eveningness and amplitude—development and validation of an improved composite scale to measure circadian preference and stability (MESSi). Chronobiol Int (2016) 33(7):832–48 [online]. Available from: 10.3109/07420528.2016.1171233 10.3109/07420528.2016.1171233 27123963

[21] Van Someren EJW Swaab DF Colenda CC Cohen W McCall WV Rosenquist PB Bright light therapy: improved sensitivity to its effects on rest-activity rhythms in Alzheimer patients by application of nonparametric methods. Chronobiol Int (1999) 16(4):505–18. 10.3109/07420529908998724 10442243

[22] Gonçalves BSB Adamowicz T Louzada FM Moreno CR Araujo JF A fresh look at the use of nonparametric analysis in actimetry. Sleep Med Rev (2015) 20:84–91. [online]. Available from: 10.1016/j.smrv.2014.06.002 10.1016/j.smrv.2014.06.002 25065908

[23] Monk TH Buysse DJ Kennedy KS Pods JM DeGrazia JM Miewald JM Measuring sleep habits without using a diary: the sleep timing questionnaire. Sleep (2003) 26(2):208–12. 10.1093/sleep/26.2.208 12683481

[24] Carney CE Buysse DJ Ancoli-israel S Edinger JD Krystal AD Lichstein KL The Consensus Sleep Diary: standardizing prospective sleep self-monitoring. Sleep (2012) 35(2):287–302. 10.5665/sleep.1642 22294820 PMC3250369

