# Supplementary material for: Use of the Pittsburgh Sleep Quality Index in People With Schizophrenia Spectrum Disorders: A Mixed Methods Study
Source: Front Psychiatry. 2019 May 9;10:284. doi: 10.3389/fpsyt.2019.00284 (PMC6520598; doi:10.3389/fpsyt.2019.00284)
Supplement: File S1 — Question schedule and optional prompts. [file DataSheet_1.docx]

Supplementary Material

The Pittsburgh Sleep Quality Index (PSQI) is a poor measure of self-reported sleep disturbance in people with schizophrenia spectrum disorders: a mixed methods study

**Sophie Faulkner^1,2^*, Chris Sidey-Gibbons^3,4^**

*** Correspondence:** Sophie Faulkner: [sophie.faulkner@manchester.ac.uk](mailto:sophie.faulkner@manchester.ac.uk)

# Supplementary Material 1: Question schedule and optional prompts

- How would you describe your sleep?

*What is that like for you?*

*Would you say that you have a ‘sleep problem’?*

- Can you tell me what it’s like when you have a poor night’s sleep?

*What’s it like getting to sleep? Being asleep? …and waking up?*

*What is it that makes this poor sleep?*

*Is there anything that impacts negatively on your sleep?*

- What would you say really good sleep is like?

Can you tell me what it’s like when you have a really good night’s sleep?

*What was it like falling asleep? Being asleep? …and when you woke up?*

*What was it about that night’s sleep that makes it ‘good sleep’?*

- Is there anything that contributes to you having good sleep?

*About the day before? About your bedtime routine? About how you’re feeling? What do you think generally affects people’s sleep? Does that affect you?*

- How important is it to you to get good sleep?

*Compared to some other things, how important is sleep? (examples?)*

- Would you say that anything in your life is affected by your sleep?

*Anything about how you feel / act / roles + tasks / physical or mental health?*

*Can you tell me about a time when something was affected by your sleep?*

- How likely would you be to seek treatment to improve any issues you have described now?
  - Where would you seek help with these issues?
- Which of these areas you rated on the PSQI would you want to change?

*How did you find completing the PSQI?*

*Is there anything you think it should have asked about that it didn’t?*

- Is there anything you have tried which helped your sleep? *(how did it help?)*
  - Is there anything anyone else has suggested, or done, which helped (friend, family, healthcare professional, book, internet or TV)? *(how did it help?)*
- Is there anything you have tried / anyone else has tried, that didn’t help?

*Why do you think that wasn’t helpful for you?*

Sleep interventions cards:

(cards with a range of existing sleep interventions and self-help strategies presented and discussed)

- Which ones do you think would be most likely to work?

*For you? For others? And why?*

- Which would be the easiest, and which would be the most difficult to use?

*why?*

- How long would you expect X to take to work?
- How would you judge if X was working?
- How likely do you think it is that your sleep will improve in future?
- Anything else you want to add?
